# Supplementary material for: Mid-Infrared Spectroscopy as a New Tool for Ruling Out Spontaneous Bacterial Peritonitis: A Proof-of-Concept Study
Source: Biomedicines. 2023 Mar 9;11(3):838. doi: 10.3390/biomedicines11030838 (PMC10045833; doi:10.3390/biomedicines11030838)
Supplement: Supplementary file 1 [file biomedicines-11-00838-s001.zip › biomedicines-2205702-supplementary.pdf]

biomedicines MDPI

Table S1. Characteristics of the patients in the calibration and validation groups.

Variable	Calibration Group (n = 170)	Validation Group (n = 86)	p
Ascites score #	3 [2–3]	3 [2–3]	0.77050
Encephalopathy score #	1 [1–1]	1 [1–1]	0.66830
Prothrombin time (%) #	56.5 [44.75–71]	58 [50–73]	0.32005
Albumin (g/L) #	28 [25–30]	29 [27–32]	0.25304
Bilirubin (µmol/L) #	44.5 [20.75–131.25]	38 [19–72]	0.35462
Blood platelet count (G/L) #	112 [69–178]	136 [94–185]	0.14113
Blood creatinine (µmol/L) #	65 [52–106]	84 [62–126]	0.06854
Age (years) #	59 [51–67]	62 [51.5–70]	0.43211
% males #	71	71	1
PMN leukocyte count (/mm^3)	9 [2.25–56]	18 [2–99.5]	0.43253
% with SBP (PMN ≥ 250/mm^3)	18	19	1
Positive ascites fluid culture (%)	11	14	0.54617
Distribution according to recruitment centers (Brest/Hyères/Monaco/Nice/Paris) %	5/7/2/33/53	5/15/0/28/52	0.16856

data from the PHRC cohort only (n = 123). Quantitative data are expressed as the median [interquartile range]. SBP: Spontaneous bacterial peritonitis; PMN: polymorphonuclear.

Table S2. Characteristics of the "false-negative" patients in the calibration and validation groups.

Sample	Group	Center	PMN Leukocyte Count (/mm^3)	Positive Ascites Fluid Culture	Prothrombin Time (%)	Albumin (g/L)	Bilirubin (µmol/L)	Blood Platelet Count (G/L)	Blood Creatinine (µmol/L)	Age
1	Calibration	Nice	483	No	65	33	38	153	114	NA
2	Calibration	Monaco	3120	No	64	30	36	82	56	63
3	Calibration	Paris	1794	No	NA	NA	NA	NA	NA	NA
4	Validation	Paris	787	No	NA	NA	NA	NA	NA	NA
5	Validation	Brest	3375	Yes	41	29	65	73	115	64

PMN: polymorphonuclear; NA: not applicable.

Table S3. Comparisons of rapid diagnostic tests for SBP (according to the literature and the present results).

	Prevalence of SBP	Se [95%CI]	Sp [95%CI]	PPV [95%CI]	NPV [95%CI]
Mid-infrared spectroscopy (the current study)	18.4% [47/256]	87% [69–100]	80% [70–88]	50% [39–65]	96% [92–100]
Reagent strip with a photospectrometer [23]	8% [12/157]	100% [70–100]	93% [87–96]	55% [33–75]	100% [97–100]
Multistix 8 SG reagent strip [16]	5.5% [117/2123]	45.3% [36.6–54.3]	99.2% [98.8–99.6]	77.9% [68.1–87.8]	96.9% [96.1–97.6]
Periscreen strip, using the "Trace" thresholds [17]	5.99% [84/1402]	91.7% [83.7–95.9]	57.1% [54.4–59.8]	12% [10.3–13.7]	99.1% [98.6–99.6]

80th percentile for calprotectin [20]	15.3% [36/236]	86.1% [70.5–95.3]	92% [87.3–95.3]	65.9% [50.7–79.1]	97.3% [93.9–99.1]
Ascitic fluid lactoferrin, cut-off at 242 ng/mL [21]	10.1% [22/218]	95.5% [77.2–99.9]	97% [93.5–98.9]	ND	ND
TREM-1, cut-off at 1199 pg/mL [11]	16% [54/337]	93% [77–99]	97% [93–100]	ND	ND

Se: sensitivity; Sp: specificity; PPV: positive predictive value; NPV: negative predictive value; CI: confidence interval; ND: not determined; TREM-1: triggering receptor expressed on myeloid cells 1.

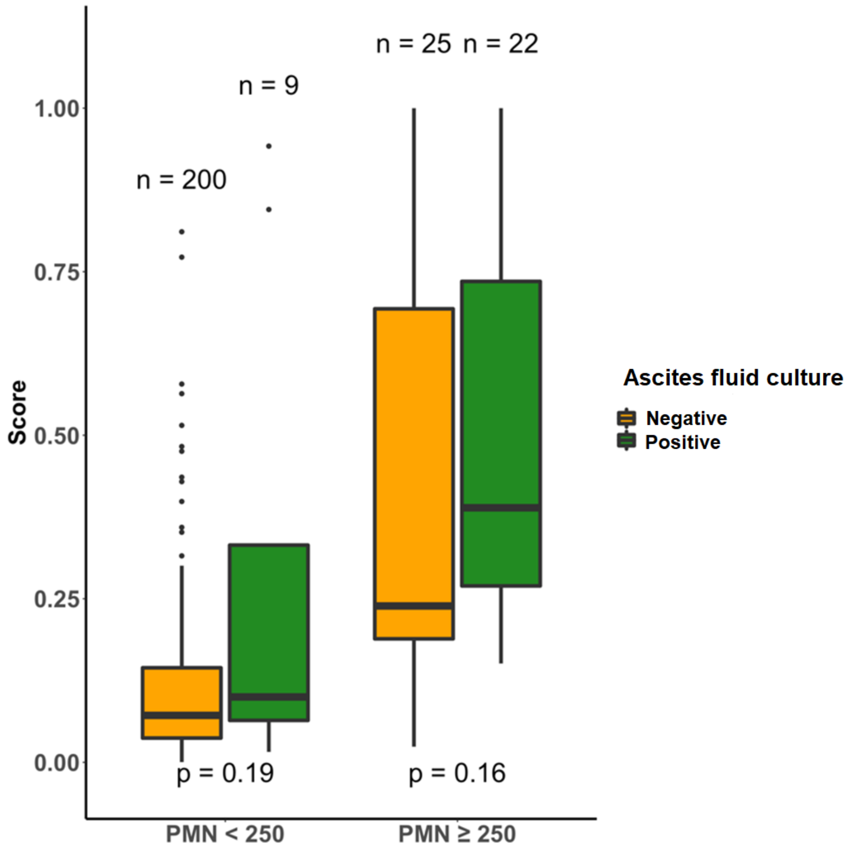

Figure S1. Comparison of the spectral model scores, according to the diagnosis of SBP (PMN <250/mm^3 vs. ≥250/mm^3) and the result of the ascites fluid culture. PMN: polymorphonuclear leukocyte count.
